# Cost-Effectiveness of Early Versus Late Interventional Radiology Drainage in Necrotizing Pancreatitis: A Decision Analysis Based on Cost Utility and Budget Impact

**DOI:** 10.7759/cureus.86152

**Published:** 2025-06-16

**Authors:** Rami Ahmad, Osborne P Vaz, Laken Boochoon, Maria Korontzi, Caroline Wolstenholme, Rami Obeidallah

**Affiliations:** 1 General Surgery, East Lancashire Teaching Hospitals NHS Trust, Blackburn, GBR; 2 Upper Gastrointestinal and General Surgery, Warrington and Halton Teaching Hospitals NHS Foundation Trust, Cheshire, GBR; 3 General Medicine, East Lancashire Teaching Hospitals NHS Trust, Blackburn, IRL; 4 General Surgery, Hospital Center Private Du Montgardé, Aubergenville, GBR; 5 General Medicine, Lancashire and South Cumbria NHS Foundation Trust, Preston, GBR; 6 General Surgery, East Lancashire Teaching Hospitals NHS Trust, Backburn, GBR

**Keywords:** acute necrotizing pancreatitis, decision analysis, incremental cost-effectiveness ratio, percutaneous catheter drainage, quality-adjusted life years

## Abstract

Introduction: The optimal timing of interventional radiology (IR) drainage in patients with necrotizing pancreatitis remains uncertain. This study compares the cost-effectiveness of early (five to six weeks) vs. late (> six weeks) IR drainage using a decision analysis model.

Methods: A retrospective cohort of 76 patients with severe necrotizing pancreatitis (2017-2021) was screened. Twenty-two patients met the inclusion criteria and were included in a decision analysis model; 11 underwent early IR drainage and 11 underwent late IR drainage. Costs, quality-adjusted life years (QALYs), and incremental cost-effectiveness ratios (ICERs) were calculated. A budget impact analysis was also conducted.

Results: Early IR drainage was associated with shorter ICU stays (mean 17 vs. 26 days, p=0.01) and fewer IR drainage sessions (median 12 vs. 28, p=0.04) compared to late drainage. Readmissions were fewer in the early group (four vs. eight; p=0.31), although this difference was not statistically significant. Rates of surgery, including ischemia or fistula, disconnected pancreatic duct syndrome, and bleeding complications, were comparable between groups. Total costs were lower in the early drainage group (£23,533-£48,526) vs. the late group (£29,168-£56,110), with slightly higher QALYs (1.77 vs. 1.76 years). The ICER for early drainage was £15,340.96 per QALY gained, within accepted UK willingness-to-pay thresholds. The budget impact analysis projected annual healthcare savings of £75,835 with early intervention.

Conclusion: In this small decision analysis, IR drainage at five to six weeks demonstrated cost-effectiveness advantages, with significantly shorter ICU stays, fewer drainage procedures, reduced costs, and similar complication rates compared to drainage after six weeks. Larger prospective studies are needed to validate these findings and guide clinical practice.

## Introduction

Severe acute pancreatitis (AP) is a life-threatening disease following a natural course consisting of early severe AP and late severe AP. The management of infected necrotizing pancreatitis is associated with prolonged hospital stays and high costs [1]. Antibiotics are administered, and intervention is postponed as long as possible in cases of proven or suspected infected pancreatic necrosis in the initial four weeks after onset of disease [2], but it is a worldwide practice for expectant management after the initial four weeks as well in stable or mildly septic patients. However, antimicrobial therapy to reduce systemic illness from the infected necrosis may lead to increased incidence of *Candida *infections and antibiotic resistance [3].

Interventional procedures are generally only performed in cases of suspected or confirmed infection of pancreatic necrosis or peripancreatic necrosis alone [2].

It is unclear whether mildly ill patients with sterile necrosis and a degree of systemic toxicity should undergo any form of invasive therapy, including debridement. It is credible, however, that inpatients with sterile necrosis who clinically deteriorate despite aggressive supportive therapy may benefit from percutaneous catheter drainage [1, 4, 5]. An international survey among expert pancreatologists has demonstrated “equipoise” between immediate and postponed catheter drainage of infected necrotizing pancreatitis [6].

Evidence of benefits from early intervention has started accumulating in recent literature: in a published systematic review of percutaneous catheter drainage as primary treatment for necrotizing pancreatitis, it was found that no additional surgical necrosectomy was required after percutaneous catheter drainage in 55.7% [7]. The Patients With Infected Necrotizing Pancreatitis (POINTER) trial is the first randomized controlled trial designed to determine the optimal timing of catheter drainage in infected necrotizing pancreatitis [8].

To the best of our knowledge, only a few studies have been conducted to compare clinical experience with primary CT-guided percutaneous catheter drainage in patients with sterile necrosis with the same drainage procedure in patients with infected necrosis. The effect of clinical comorbid conditions, such as multisystem organ failure, on the outcome of primary CT-guided percutaneous catheter drainage in patients with sterile necrosis has not been assessed.

Sugimoto et al. found better outcomes of early and proactive use of percutaneous drainage during the course of necrotizing pancreatitis (lower incidences of organ failure, need for necrosectomy, and in-hospital mortality) [9]. The use of a preemptive percutaneous catheter drainage protocol early, before the development of severe sepsis, appeared to be effective.

Walser et al. found that the presence of sterile necrosis did not confer a more favorable patient outcome. The patients with sterile necrosis presumably had a higher mortality since their clinical presentation was much inferior to that of patients with infected necrosis. This observation reinforces the hypothesis, validated by other investigators [10].

Our aim was to define the optimal timing of radiological intervention for abdominal collections between five to six weeks and > six weeks in patients with absent or mild organ dysfunction during the late phase of acute necrotizing pancreatitis. For this, we conducted a cost-effectiveness decision analysis using our hospital database and data from the international literature.

## Materials and methods

This retrospective study was conducted at East Lancashire Teaching Hospitals NHS Trust, Blackburn, UK, and included anonymized data from 76 patients admitted with severe necrotizing pancreatitis and abdominal collections between 2017 and 2021. Clinical and economic data such as hospital length of stay, ICU admissions, organ dysfunction, collection characteristics, procedures (radiological, endoscopic, surgical), complications, readmissions, and direct medical costs were organized using Microsoft Excel (Microsoft Corp., Armonk, NY).

A literature review from 2010 to 2021 was performed via Medical Literature Analysis and Retrieval System Online (MEDLINE), PubMed, and Cochrane Library, focusing on necrotizing pancreatitis-related terms including cystogastrostomy, image-guided drainage, cholangitis, bleeding complications, and Short Form-36 (SF-36) health survey scores. Systematic reviews and clinical guidelines were prioritized [8,11,12].

Inclusion criteria were patients aged ≥18 years diagnosed with necrotizing pancreatitis and radiologically confirmed abdominal collections, hospitalized for over four weeks, with recurrent or new collections, and initial single organ dysfunction or less. Exclusion criteria included absence of collections, drainage within four weeks of onset, interhospital transfer, multi-organ failure, known ductal disruption or minimal fluid collections [12], chronic pancreatitis, pancreatic malignancy, and death within two years of follow-up.

Interventional radiology (IR) drainage was classified as early (five weeks post-onset) or late (> six weeks). Clinical outcomes such as readmissions, number of IR sessions, ICU stay, surgeries, pancreatic duct disruption, and bleeding were compared between groups using Fisher’s exact test for categorical data and the Mann-Whitney U Test for continuous variables. These analyses informed economic modeling by identifying cost- and quality-of-life-related variables.

Quality-adjusted life years (QALYs) were the primary outcome, reflecting survival duration and quality (0 = death, 1 = perfect health). Utility values were derived from published sources [13]. The incremental cost-effectiveness ratio (ICER) was calculated as the cost difference divided by the QALY difference; a negative ICER indicates dominance. Cost-effectiveness was interpreted using UK willingness-to-pay (WTP) thresholds [13,14]. One-way sensitivity analyses assessed model robustness by varying individual parameters [13].

**Figure 1:**
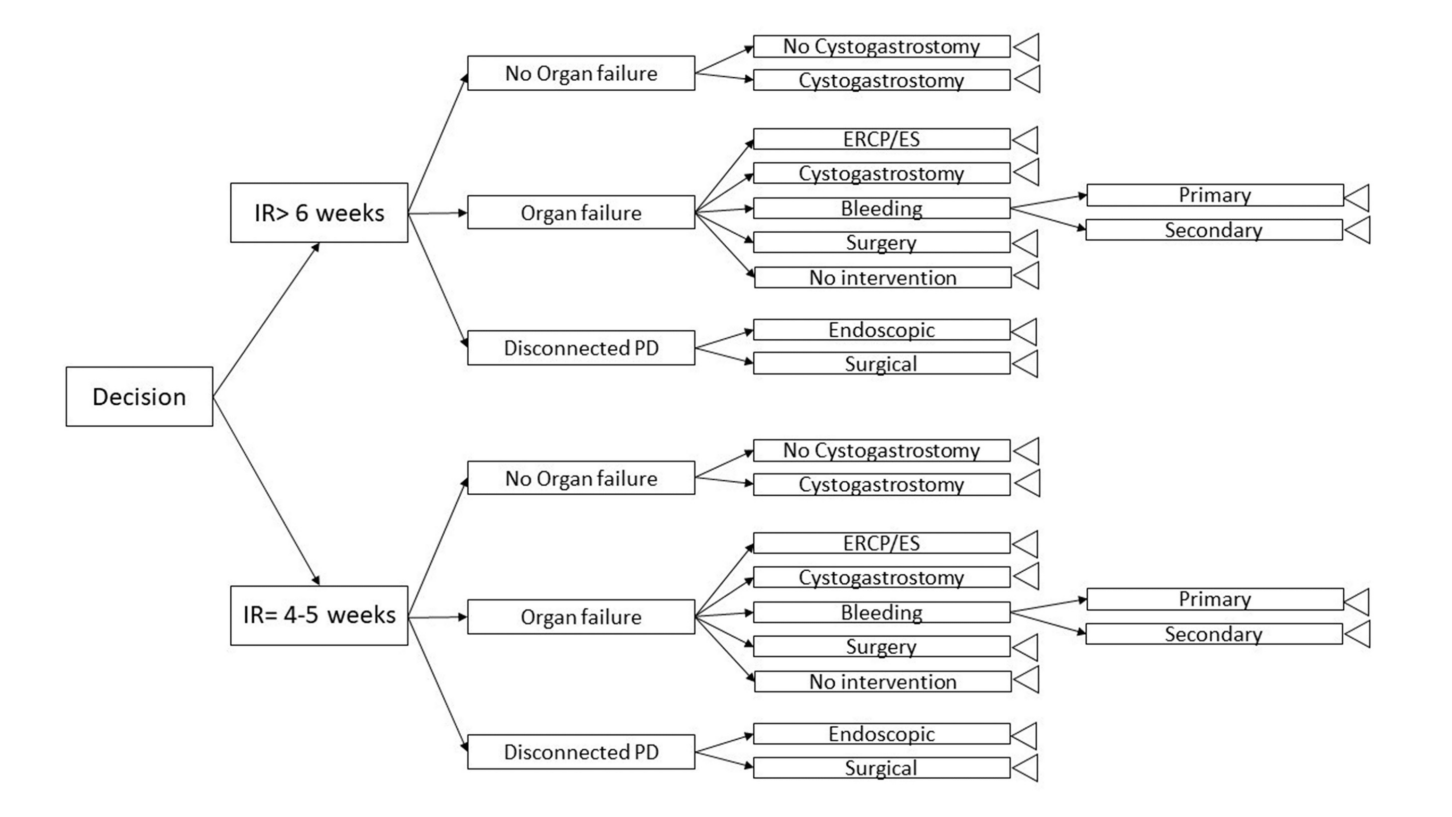
Decision analysis model to examine the cost-effectiveness of the two strategies for the optimal timing of interventional radiology (IR) procedure. PD: pancreatic duct; ERCP: endoscopic retrograde cholangiopancreatography; ES: endoscopic sphincterotomy

A decision tree was built using Decision Tree Analyzer v3.7.1 (Spicelogic Inc., Ontario, Canada), incorporating clinical and economic outcomes (Table 1, Figure 1).

**Table 1:** Independent predictors of the medical costs from our patient cohort. Test: Generalized linear/nonlinear models (method: sigma-restricted parametrization) *IR sessions: interventional radiology session to apply percutaneous catheter drainage for abdominal collections

Parameters	Estimate	Standard error	Wald statistic	p-value
Intercept	7.08673	0.045721	24025.22	<0.001
Number of IR sessions*	-0.15453	0.010102	233.98	<0.001
Days of first IR since onset	0.00073	0.000269	7.39	0.006
Number of readmissions	0.99740	0.008999	12285.54	<0.001
System dysfunction	-3.29953	0.033770	9546.53	<0.001
Endoscopy	0.08727	0.014958	34.04	<0.001
Bleeding	-1.51307	0.014049	11599.66	<0.001
Disconnected pancreatic duct	-1.80792	0.021621	6992.29	<0.001
Surgery/Bowel ischaemia/fistula	0.22059	0.011676	356.93	<0.001

The model compared IR drainage timing groups, accounting for possible clinical courses including organ failure, duct syndrome, cystogastrostomy, endoscopic retrograde cholangiopancreatography (ERCP), bleeding, surgery, or conservative recovery. Outcome probabilities were based on cohort data and literature.

QALYs were calculated from SF-36 Physical Component Summary (SF-36 PCS) scores across three intervals: 0 to four months, four to 12 months, and 12 to 24 months [15-17]. Cost data per patient and intervention are summarized in Tables 2, 3.

**Table 2:** Cost values for activity per patient used in the model N: number of patients; all costs are in British pounds (GBP, £); CNST: Clinical Negligence Scheme for Trusts; hyphens (–) are used only to indicate missing or non-applicable data; all valid numeric values are preserved.

Activity	2017	2018	2019	2020	All year total costs (£)	N	Costs of activity per patient (£)
All other tests	3365.72	2126.93	13076.40	8273.97	26843.02	76	353.20
Anaesthesia	1944.23	1253.85	1412.94	1150.84	5761.86	76	75.81
Biochemistry	3295.75	1292.48	9558.17	5595.00	19741.40	76	259.76
Cellular sciences	118.14	4.92	474.56	411.10	1008.72	76	13.27
CNST indemnity	25435.70	20549.57	45747.47	39507.41	131240.15	76	1726.84
Dispensing blood products and factor products	2402.02	1107.47	1823.11	1165.30	6497.90	76	85.50
Dispensing high-cost drugs (on the list)	–	–	7586.93	1318.95	8905.88	76	117.18
Dispensing non-patient-identifiable medicines	3450.88	3404.74	10311.56	8799.59	25966.77	76	341.67
Dispensing high-cost drugs (duplicate entry)	–	1409.28	7143.89	2451.83	11005.00	76	144.80
Fluoroscopy	7.95	–	120.54	97.11	225.60	76	2.97
Hematology	1824.54	1850.37	5965.20	4202.45	13842.56	76	182.14
Microbiology	1080.71	449.15	348.09	233.51	2111.46	76	27.78
MRI	519.87	744.44	242.28	118.11	1624.70	76	21.38
No activity required	–	1114.32	29336.10	30826.74	61277.16	76	806.28
Diagnostic imaging: primary bleeding	6510.62	–	20057.35	3513.49	30081.46	4	4377.28
Diagnostic imaging: primary and secondary bleeding	–	–	–	16045.88	16045.88	1	16045.88
Other multidisciplinary team meetings	175.68	142.17	1489.07	1414.49	3221.41	76	42.39
Outpatient care	–	–	272.13	141.35	413.48	76	5.44
Outreach contacts	–	7114.67	8070.68	5508.53	20693.88	76	272.29
Pharmacy (other activity)	1749.71	7233.23	22217.70	18810.65	50011.29	76	658.04
Plain film	132.03	201.92	257.35	145.63	736.93	76	9.70
Readmission	16356.97	24685.00	87315.19	–	128832.00	9	14315.00
Ultrasound	387.83	1132.53	3222.40	2529.11	7271.87	76	95.68
Ward care	97269.59	98207.02	218493.22	215221.56	629191.39	76	8278.83
CT-scan	3168.11	4514.30	3439.57	2205.27	13327.25	76	175.36
Support services	1882.41	11775.38	14592.49	14107.33	42357.61	15	2823.84
Supporting contacts	7226.08	9486.92	58831.46	36405.73	111950.19	15	7463.35
Endoscopy	4176.28	3762.53	14338.65	13885.97	36163.43	27	1339.39
Prosthesis, implant, or device insertion	–	1292.48	9558.17	5595.00	16445.65	27	609.10
Surgical care	6714.20	6518.47	7065.27	6505.82	26803.76	11	2436.71

**Table 3:** Quality-adjusted life years (QALYs) over two-year follow-up *PCS: Physical Component Summary of the Short Form (SF)-36 evaluation tool

Time period	Clinical characteristics/Events	QALY value	Measurement tool	Reference
4 months	ICU stay >25 days	0.63	PCS*, SF-36	[11]
4 months	No ICU stay or ICU stay <25 days	0.76	PCS, SF-36	[11]
>4–12 months	Pain, analgesic use, readmissions, recurrent/chronic pancreatitis, diabetes	0.70	PCS, SF-36	[15]
>12–24 months	Not specified	0.80	PCS, SF-36	[15]

The model assumed all patients required IR drainage. Single-organ dysfunction occurred in ~41%, with progression to multi-organ failure included. Cystogastrostomy was anticipated in 10% to 14%, ERCP in 14% of biliary cases, bleeding complications in 6.5% (3% delayed), surgery in 14%, conservative recovery in 12.5%, and disconnected duct syndrome in ~19.7% [18]. Average readmissions ranged from 0.5 to one, and IR sessions from 1.2 to 1.9 per patient. Cost and utility inputs were drawn from institutional data and literature.

Cost-effectiveness was analyzed from a societal perspective with base-case and sensitivity analyses. A five-year budget impact analysis followed National Institute for Health and Care Excellence (NICE) digital health technology standards [19], using Mauskopf’s framework for care pathways and costs [16].

Statistical analysis predictors of total hospital costs were identified using generalized linear models (GLM) with maximum likelihood estimation. Group comparisons for clinical outcomes employed Fisher’s exact test for categorical variables and the Mann-Whitney U Test for continuous variables. Analyses were conducted using STATISTICA v7 (StatSoft Inc., Tulsa, OK).

## Results

The systematic literature review found limited studies directly comparing early and late percutaneous catheter drainage in patients with sterile necrosis or infected necrosis without significant organ dysfunction [1,7,11,20]. Cost values for individual healthcare activities per patient, based on the entire 76-patient cohort, are presented in Table 2.

Among the 76 patients, 22 met the study’s inclusion criteria and underwent image-guided IR drainage. Baseline characteristics were similar between early and late IR groups, with comparable age, gender, American Society of Anesthesiologists (ASA) grades, and comorbidities. Biliary pancreatitis was more common in the early group; alcohol-related causes were slightly higher in the late group (Table 4).

**Table 4:** Baseline characteristics of patients undergoing early vs. late IR drainage M: male; F: female; ASA: American Society of Anesthesiologists; COPD: chronic obstructive pulmonary disease; ERCP: endoscopic retrograde cholangiopancreatography; IR: interventional radiology

Characteristic	Early IR drainage (n=11)	Late IR drainage (n=11)	p-value
Age (mean ± SD)	56.2 ± 14.1	58.6 ± 13.7	0.68
Gender (M/F)	07-Apr	06-May	0.68
ASA Grade			0.81
ASA I	1	0	
ASA II	3	2	
ASA III	5	6	
ASA IV	2	3	
Comorbidities			
Diabetes mellitus	4	5	0.66
Hypertension	5	6	0.68
Ischemic heart disease	3	4	0.64
Chronic kidney disease	2	3	0.61
COPD	2	2	1
Etiology of pancreatitis			0.86
Biliary	7	5	
Alcohol-induced	3	4	
Idiopathic	1	1	
Post-ERCP	0	1	

These patients accounted for a total of 40 procedures and were evenly divided into early and late drainage groups. A decision-analysis model was employed to compare outcomes.

Patients in the early drainage group demonstrated better clinical outcomes. They had shorter ICU stays (17 vs. 26 days, p=0.01 by Mann-Whitney U Test), fewer readmissions (four vs. eight, p=0.31 by Fisher’s Exact Test), and required fewer IR drainage sessions (median 12 vs. 28, p=0.04 by Mann-Whitney U Test). Surgical intervention rates (including ischemia or fistula), rates of disconnected pancreatic duct syndrome, and bleeding complications (primary and secondary) were similar between groups, with no statistically significant differences (all p=1.00 by Fisher’s exact test). These outcomes were considered important drivers of both costs and quality of life (Table 5).

**Table 5:** Base-case values used in sensitivity analysis outcomes of early (five to six weeks) vs. late (> six weeks) IR drainage with statistical testing and impact IR: interventional radiology

Outcome	Early IR (n = 11)	Late IR (n = 11)	Statistical test	p-value	Factors impacted
Readmissions	4	8	Fisher’s exact test	0.31	Costs/Quality of life
IR sessions (median)	12	28	Mann–Whitney U test	0.04	Costs
ICU stay (mean)	17 days	26 days	Mann–Whitney U test	0.01	Quality of life
Surgery (including ischemia/fistula)	2	2	Fisher’s exact test	1	Costs/Quality of life
Disconnected pancreatic duct	3	2	Fisher’s exact test	1	Costs/Quality of life
Primary bleeding	1	2	Fisher’s exact test	1	Costs/Quality of life
Secondary bleeding	0	1	Fisher’s exact test	1	Costs/Quality of life

Over the two-year follow-up, the early group also accrued a slightly higher average QALY score (1.77 compared to 1.76 years), although the difference was modest (Table 6).

**Table 6:** Possible paths with total payoffs IR: interventional radiology; ERCP: endoscopic retrograde cholangiopancreatography; ES: endoscopic sphincterotomy; QALY: quality-adjusted life years; all costs are in British pounds (£)

Terminal	Total payoffs
IR> 6 weeks	
→ Chance → No organ failure → Chance → No cystogastrostomy	1.77 QALY; £29,168
→ Chance → No organ failure → Chance → Cystogastrostomy	1.77 QALY; £31,116
→ Chance → Organ failure → Chance → ERCP/ES	1.78 QALY; £40,794
→ Chance → Organ failure → Chance → Cystogastrostomy	1.76 QALY; £43,352
→ Chance → Organ failure → Chance → Surgery	1.76 QALY; £41,892
→ Chance → Organ failure → Chance → No intervention	1.76 QALY; £39,455
→ Chance → Disconnected duct → Chance → Endoscopic management	1.77 QALY; £31,116
→ Chance → Disconnected duct → Chance → Surgical management	1.77 QALY; £32,944
→ Chance → Organ failure → Chance → Bleeding → Chance → Primary	1.76 QALY; £44,441
→ Chance → Organ failure → Chance → Bleeding → Chance → Secondary	1.76 QALY; £56,110
IR 5-6 weeks	
→ Chance → No organ failure → Chance → Cystogastrostomy	1.77 QALY; £23,533
→ Chance → No organ failure → Chance → No cystogastrostomy	1.77 QALY; £21,584
→ Chance → Disconnected duct → Chance → Surgery	1.77 QALY; £25,360
→ Chance → Disconnected duct → Chance → Endoscopic intervention	1.77 QALY; £23,533
→ Chance 2 → Organ failure → Chance → No intervention	1.77 QALY; £31,872
→ Chance 2 → Organ failure → Chance → Surgery	1.77 QALY; £34,308
→ Chance 2 → Organ failure → Chance → Cystogastrostomy	1.77 QALY; £35,768
→ Chance 2 → Organ failure → Chance → ERCP/ES	1.77 QALY; £33,211
→ Chance 2 → Organ failure → Chance → Bleeding→ Chance → Secondary	1.76 QALY; £48,526
→ Chance 2 → Organ failure → Chance → Bleeding → Chance → Primary	1.76 QALY; £36,858

The average cost per patient was lower in the early drainage group, ranging from £23,533 to £48,526, compared to £29,168 to £56,110 in the late group (Table 6). The ICER for early drainage was calculated as £15,340.96 per QALY gained, which falls within the accepted WTP range in the UK of £20,000 to £30,000 per QALY (Table 7).

**Table 7:** Cost-effectiveness comparison between early (five to six weeks) and late (> six weeks) interventional radiology (IR) drainage in necrotizing pancreatitis

Metric	Early IR drainage	Late IR drainage	Increment/difference
Average cost per patient (£)	£23,533 – £35,768	£29,168 – £56,110	Early is £5,635 – £20,342 less costly
Quality-adjusted life years (QALYs)	1.77 years	1.76 years	+0.01 QALYs (slight improvement)
Incremental cost-effectiveness ratio (ICER)	–	–	£15,340.96 per QALY gained (early vs. late)

Multivariate regression identified several independent predictors of increased cost (Table 1). These included a higher number of IR drainage procedures, delayed drainage, an increased number of readmissions, the presence of organ dysfunction, the use of endoscopic interventions (including cystogastrostomy and necrosectomy), bleeding complications, duct disruption requiring stenting, and surgical interventions for bowel ischemia or fistula. Each of these factors contributed significantly to both the economic and clinical burden.

The budget impact analysis was based on the local population of East Lancashire, estimated at 377,111 people. With an annual pancreatitis incidence of 0.06%, approximately 207 patients were expected each year, of whom 30% would develop necrotizing pancreatitis. An estimated 16 of these patients would qualify for early IR drainage annually (Table 8).

**Table 8:** Eligible population and uptake over time IR: interventional radiology

Year	Current period (Year 0)	2022/23	2023/24	2024/25	2025/26	2026/27
Eligible population	16	16	16	16	16	16
Uptake of IR drainage at five to six weeks (%)	100%	100%	100%	100%	100%	100%
Number of people using new technology	16	16	16	16	16	16

Implementing early drainage in this group was projected to result in annual healthcare savings of £75,835 (Tables 9-11)

**Table 9:** Cost components of current practice (per year) Total cost of current practice (£) 390,388; ERCP: endoscopic retrograde cholangiopancreatography; ES: endoscopic sphincterotomy

Pathway description	Cost (£)
No organ failure → Cystogastrostomy	29,168
No organ failure → No cystogastrostomy	31,116
Disconnected duct → Surgery	40,794
Disconnected duct → Endoscopic	43,352
Organ failure → No intervention	41,892
Organ failure → Surgery	39,455
Organ failure → Cystogastrostomy	31,116
Organ failure → ERCP/ES	32,944
Organ failure → Bleeding → Secondary intervention	44,441
Organ failure → Bleeding → Primary intervention	56,110

**Table 10:** Cost components of future practice (per year) Total cost of future practice (£) 314,553; ERCP: endoscopic retrograde cholangiopancreatography; ES: endoscopic sphincterotomy

Pathway description	Cost (£)
No organ failure → Cystogastrostomy	23,533
No organ failure → No cystogastrostomy	21,584
Disconnected duct → Surgery	25,360
Disconnected duct → Endoscopic	23,533
Organ failure → No intervention	31,872
Organ failure → Surgery	34,308
Organ failure → Cystogastrostomy	35,768
Organ failure → ERCP/ES	33,211
Organ failure → Bleeding → Secondary intervention	48,526
Organ failure → Bleeding → Primary intervention	36,858

**Table 11:** Total costs and net budget impact over time

Year	2022/23	2023/24	2024/25	2025/26	2026/27
Total cost of current practice (£)	3,90,388	3,90,388	3,90,388	3,90,388	3,90,388
Total cost of future practice (£)	3,14,553	3,14,553	3,14,553	3,14,553	3,14,553
Net budget impact (£)	-75,835	-75,835	-75,835	-75,835	-75,835

**Figure 2:**
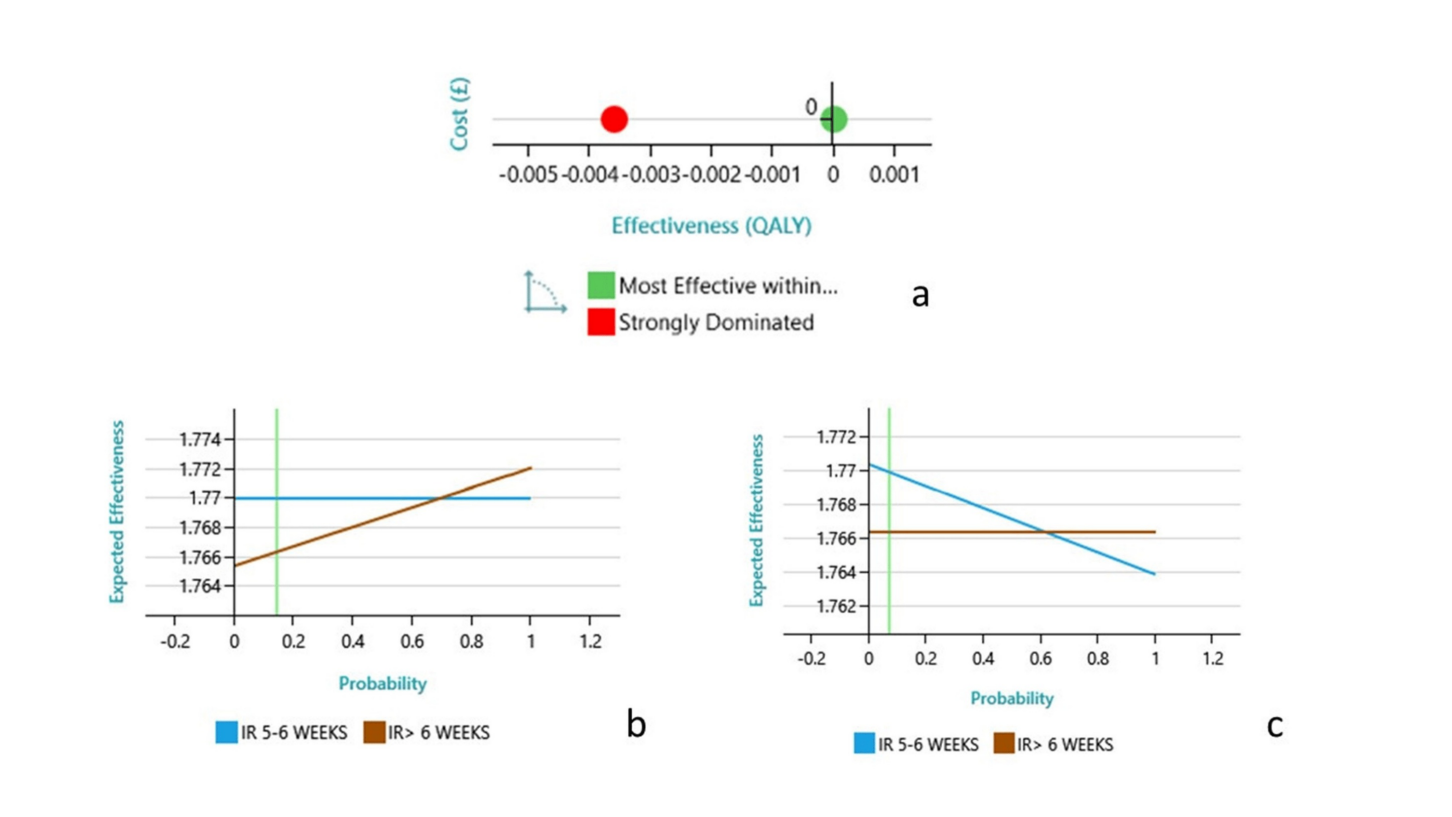
(a) Interventional radiology (IR) drainage within five to six weeks at the late phase of necrotizing pancreatitis is the preferred cost-effective option. Shifting cost-effectiveness towards the IR > six weeks option, there is a need for 70% of patients (probability 0.7) to have an early (endoscopic retrograde cholangiopancreatography) (ERCP) (for biliary pancreatitis) (b), or if bleeding is present in 60% of patients (c). The vertical green line in panels b and c indicates the base-case probability value used in the deterministic model. It serves as a reference point to assess how variations in the input probability influence the expected effectiveness of the two strategies. QALY: quality-adjusted life years

The decision-analysis model integrated empirical clinical data with real-world cost estimates and probabilities for clinical events. Early drainage consistently emerged as the dominant strategy across most scenarios with favorable clinical outcomes and economic efficiency. Visual representation of model outcomes is provided in Figure 2.

However, as shown in Figure 2, the one-way sensitivity analysis revealed two factors that could influence this outcome: if more than 70% of patients required early ERCP for biliary pancreatitis or if the bleeding complication rate exceeded 60%, late drainage could become more favorable. 

Analysis of clinical events revealed that 13 patients (59%) experienced organ or system dysfunction. Endoscopic cystogastrostomy with necrosectomy was performed in three patients (across six sessions), and ERCP was conducted in 10 patients. Four patients experienced bleeding (three primary, one secondary), while five patients were diagnosed with disconnected pancreatic duct syndrome. Four patients required surgical intervention for bowel ischemia or fistula. In total, 12 patients were readmitted after discharge. These clinical events significantly influenced both outcomes and costs (Table 12).

**Table 12:** Clinical events and interventions among study population (n = 22) ERCP: endoscopic retrograde cholangiopancreatography

Clinical event/Intervention	Number of patients	Notes/Details
Organ/system dysfunction	13	59% of total patients
Endoscopic cystogastrostomy with necrosectomy	3	Total of six sessions
ERCP performed	10	Includes both diagnostic and therapeutic
Bleeding complications	4	Three primary bleeds, one secondary delayed
Pancreatic duct disruption requiring transpapillary stenting	5	-
Surgical intervention (bowel ischemia or fistula)	4	-
Readmission post-discharge	12	-

## Discussion

The aim of our decision analysis was to determine the optimal timing for IR percutaneous catheter drainage in patients with sterile pancreatic necrosis or infected necrosis with or without single organ dysfunction during the late phase of acute necrotizing pancreatitis. Our findings indicate that IR drainage at five to six weeks is more cost-effective than later drainage (> six weeks), offering both lower costs and slightly improved QALYs. The presence of a cost-effectiveness frontier for drainage at five to six weeks makes this timing an optimal choice, influenced mainly by biliary etiology rates and risks of bleeding. These conclusions remained robust across a wide range of model assumptions; for example, even a bleeding rate as high as 60% would not negate the cost-effectiveness of earlier drainage [21].

To the best of our knowledge, this is the only study that addresses the cost-effectiveness of CT-guided percutaneous catheter drainage in sterile and mildly infected necrosis within a late phase of the acute necrotizing pancreatitis population using a robust decision-analysis model.

Our analysis aligns with broader recommendations from the Institute for Clinical and Economic Review [22], which advocates combining (1) a systematic review of comparative effectiveness and (2) a de novo decision model informed by base-case parameters. We adhered to these principles, supplementing the literature where gaps existed with predictors derived from our own real-world clinical dataset.

Several recent studies further support the clinical and strategic relevance of our analysis. Van Veldhuisen et al., 2024 [23], compared early versus late drainage in necrotizing pancreatitis and emphasized improved patient-centered outcomes such as reduced complications and faster recovery in the early group. Similarly, findings from the POINTER trial (Boxhoorn et al., 2021) [24] have reinforced the relevance of timely, step-up interventions, especially where delayed drainage may lead to progression of sepsis or complications. These studies highlight the need for individualized, early interventions, which our economic analysis now validates from a cost-effectiveness and budget impact perspective.

From a health economics standpoint, our model suggests that decision-making for IR drainage should incorporate ICER thresholds, biliary disease burden, and anticipated bleeding risk. The analysis confirms that drainage at five to six weeks is not only clinically sound but also financially advantageous under the NHS framework.

Additionally, budget impact analysis (BIA) is essential alongside cost-effectiveness modeling. It estimates the financial implications of adopting early drainage strategies across NHS trusts, especially when considering the potential transfer of patients from district general hospitals. BIA supports real-world implementation by mapping costs to current service delivery constraints [13].

Our use of net benefit analysis confers advantages: it provides a direct comparison across strategies regardless of the number of alternatives, and it reduces the instability often associated with ICERs in scenarios where differences in effect size are small [25].

Our study has several limitations. Although we analyzed clinical outcomes, we did not include genetic risk factors such as SPINK1, IL-1β, or IL-10 mutations, which may influence disease severity [26]. The study was single-center with a relatively small sample size, limiting external generalizability. Costs from district general hospitals were not included for transferred patients. We did not evaluate health opportunity costs. The impact of pancreatitis on mental health and quality of life, especially in alcohol-related cases, was not considered due to a lack of robust published data. Finally, long-term morbidity costs, including follow-up for pseudocyst, endocrine, or exocrine insufficiency, were not incorporated.

## Conclusions

In summary, early IR drainage performed at five to six weeks in necrotizing pancreatitis appears to be a cost-effective approach. It was associated with shorter ICU stays, fewer procedures, and lower overall costs, with comparable complication rates and a slight improvement in QALYs. The ICER was within accepted UK thresholds, and the budget impact analysis projected significant annual savings.

Although the sample size was limited and the study was conducted at a single center, the findings align with emerging clinical evidence supporting early intervention. Larger, prospective studies are needed to confirm these results and guide practice. Overall, early IR drainage may offer both clinical and economic advantages in appropriate patients.
